# Innovation in endocrine surgery: robotic versus laparoscopic adrenalectomy. Meta-analysis and systematic literature review

**DOI:** 10.18632/oncotarget.22059

**Published:** 2017-10-19

**Authors:** Antonino Agrusa, Giorgio Romano, Giuseppe Navarra, Giovanni Conzo, Gianni Pantuso, Giuseppe Di Buono, Roberto Citarrella, Massimo Galia, Attilio Lo Monte, Gaspare Cucinella, Gaspare Gulotta

**Affiliations:** ^1^ Department of General Surgery, Urgency and Organ Transplantation, University of Palermo, Palermo, Italy; ^2^ Department of Human Pathology University Hospital of Messina, Messina, Italy; ^3^ Department of Anesthesiologic, Surgical and Emergency Sciences, Division of General and Oncologic Surgery, School of Medicine, Second University of Naples, Naples, Italy; ^4^ Department of Surgery and Oncology, University of Palermo, Palermo, Italy; ^5^ Department of Experimental Biomedicine and Clinical Neurosciences, University of Palermo, Palermo, Italy; ^6^ Section of Radiology - Di.Bi.Me.F., University of Palermo, Palermo, Italy; ^7^ Department of Obstetrics and Gynecology, University of Palermo, Palermo, Italy

**Keywords:** laparoscopic adrenalectomy, robotic adrenalectomy, laparoscopic surgery, robotic surgery, adrenal surgery

## Abstract

Several studies in the last years demonstrated the better surgical outcome of laparoscopic approach to adrenal gland. Laparoscopic surgery is more difficult to learn and requires different psychomotor skills than open surgery, especially with regard to complex maneuvers requiring precision and dexterity. The development of robotic platform with three-dimensional vision and increased degrees of freedom of the surgical instruments has the aim to overcome these problems. We performed a systematic literature review with meta-analysis to evaluate preoperative data and surgical outcomes of robotic adrenalectomy compared with laparoscopic technique. In September 2016 we performed a systematic literature review using the Pubmed, Scopus and ISI web of knowledge database with search term “robotic adrenalectomy”. We identified 13 studies with eligible criteria that compared surgical outcomes. This present systematic review with meta-analysis includes 798 patients: 379 underwent to robotic adrenalectomy (cases group) and 419 to laparoscopic adrenalectomy (controls group). There were no significant differences between the two groups of patients respect to age, gender, laterality and tumor size. BMI instead was significant lower in the robotic group. In this group we found also patients with higher incidence of previous abdominal surgery. The results from operative time demonstrated lower operative time for laparoscopic group but there were no significant differences with robotic group. Robotic adrenalectomy showed a significant lower blood loss. Robotic adrenalectomy is a safe and feasible technique with reduced blood loss and shorter hospital stay than laparoscopic adrenalectomy. Laparoscopic approach seems to be a more rapid technique when comparing to robotic technique, although recent studies demonstrate a significant operative time reduction in robotic group with the learning curve improvement and the development of new surgical technology.

## INTRODUCTION

Since the first description by Gagner in 1992 [1] laparoscopic adrenalectomy (LA) became the standard treatment for adrenal removal. Several studies in the last years demonstrated the better surgical outcome of laparoscopic approach to adrenal gland such as decrease of the perioperative morbidity, lower complication rate, less operative blood loss, less perioperative pain and short hospital stay compared with open adrenalectomy for several indications [2–5]. Some questions remain about treatment of large adrenal masses with increased risk of adrenocortical carcinoma (ACC) [6]. Laparoscopic surgery is more difficult to learn and requires different psychomotor skills than open surgery. In fact, the surgeons have to work in a three-dimensional space, but are guided by two-dimensional images. This limitation can be challenging, especially with regard to maneuvers requiring precision and dexterity [7]. For this reason in the last period the use of a new generation three-dimensional (3D) HD laparoscopic system can improved quality of vision [8, 9, 10], but as the current laparoscopic technology is limited in regard to the maneuverability and inferior ergonomic design of instruments, natural surgical strain, tremors and the counterintuitive movements. The development of robotic platform has the scope to overcome these problems. In 1999 Piazza et al. [11] published the first case of robot-assisted right adrenalectomy in a patients with Conn's syndrome using the ZEUS AESOP (Computer Motion, Inc., Santa Barbara, CA). With the introduction of the da Vinci system (Intuitive Surgical, Sunnyvale, CA, USA) several series of robotic surgery have been reported. In recent years robotic adrenalectomy (RA) has received attention to the perceived benefits of this technology with three-dimensional vision, the elimination of surgeon's tremor and increased degrees of freedom of the surgical instruments, a comfortable sitting position. Nonetheless, robotic surgical operations seem to have longer operative time and more expensive costs compared with traditional laparoscopic surgery. In recent years several authors described cases series of robotic approach to adrenal surgery compared with traditional LA and demonstrated safety and feasibility of robotic procedure with different results in term of operative time, blood loss, conversion rate, complications and length of hospital stay. Nevertheless, these reports did not consider short and long-term outcomes so we performed a systematic literature review with meta-analysis to compare preoperative differences and surgical outcomes between RA and LA.

## MATERIALS AND METHODS

### Literature search and study selection

In September 2016 we performed a systematic literature review using the Pubmed, Scopus and ISI web of knowledge database to identify all studies that compared RA and LA. The search was done using the term “robotic adrenalectomy”. We considered only publications in English language that compared robot-assisted and laparoscopic adrenalectomy. Conference abstracts, cases series, non comparative studies and comparison with open surgery were not included in this meta-analysis. When two or more studies were published by the same authors and/or institution with a potentially overlapping patient sample and with the same outcome we considered the most recent. Three independent reviewers completed this process with analysis of cited references from the selected articles to identify other significant articles.

### Study quality assessment

**Figure 1:**
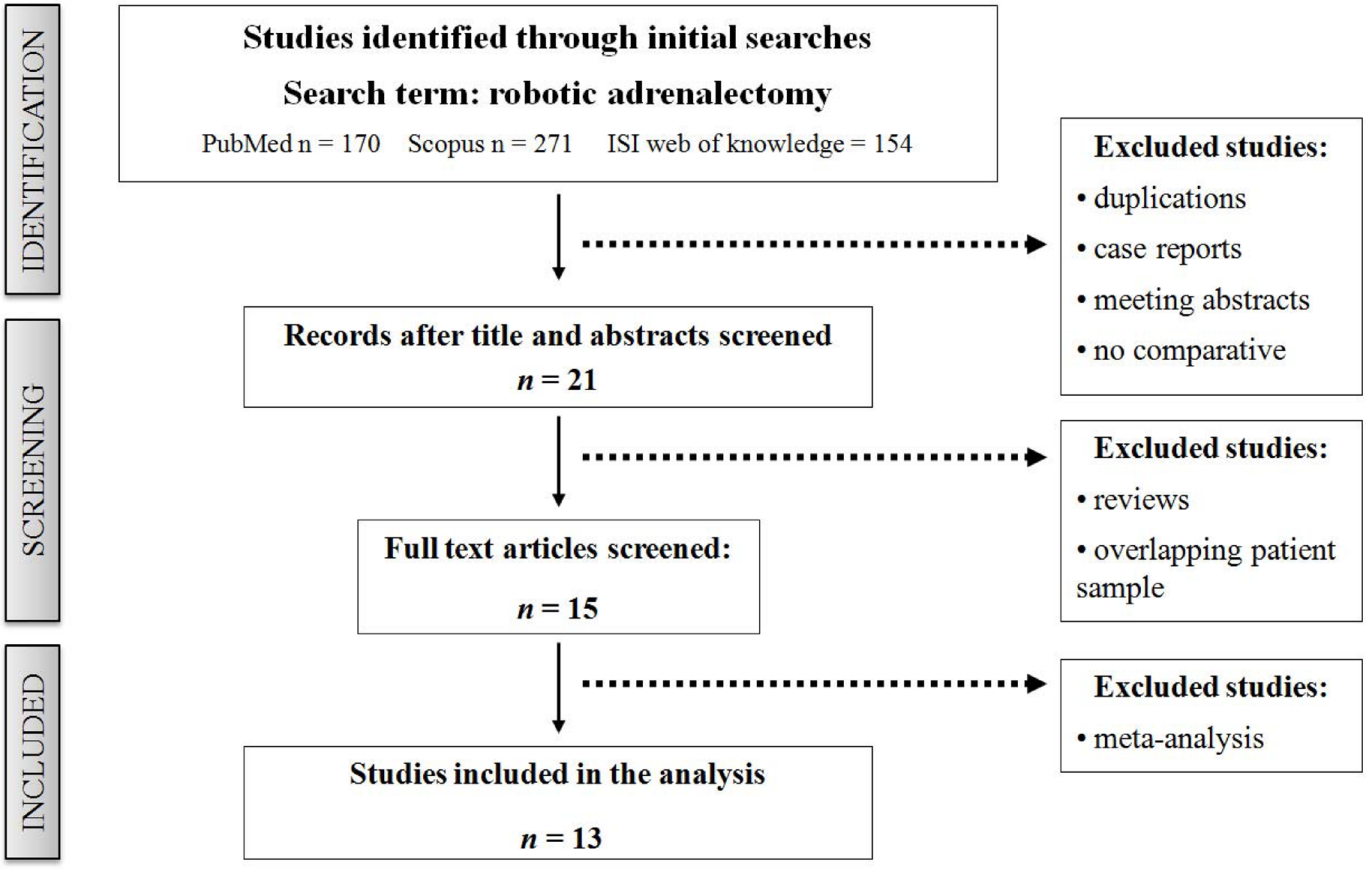
Systematic literature review to identify all studies that compared RA and LA

The level of evidence of included studies was rated according to the criteria of the Centre for Evidence-Based Medicine [12]. The methodological quality of all nonrandomized studies was assessed using the Newcastle-Ottawa Scale [13]. A score of 0–9 may be given to individual studies. Studies achieving a score of 7 or more indicated a higher quality. Three reviewers (AA, GR and GDB) independently assessed the quality of the study and solved disagreement by consensus. We identified 13 studies [14–26] with eligible criteria (Figure 1) that compared RA versus LA and clinical outcomes.

### Data extraction and outcomes of interest

The clinical outcomes that were analyzed and compared among RA and LA included preoperative demographic characteristics like age, gender, body mass index (BMI); tumors size and laterality, surgical indication and pathology specimens; history of previous abdominal surgery; specific surgical approach to adrenal gland (lateral transperitoneal versus retroperitoneoscopic). Surgical results such as operative time, blood loss, conversion rate, complications and length of hospital stay were considered indirect objective signs of surgical precision and safety.

### Statistical analysis

A meta-analysis was performed to identify clinical outcomes and potentially surgical advantages of RA when compared with traditional LA. This analysis follows the recommendations of the Cochrane Collaboration using R Statistical Software (R ver 3.3.1, R Foundation for Statistical Computing, Vienna, Austria). Odds ratio (OR) was used for dichotomous variables and mean difference or standardized mean difference for the continuous parameters. All outcomes were reported with 95% confidence interval (95% CI). However some studies did not report any of these parameters, but presented continuous data as medians. In these cases we made an approximate transformation using Hozo methodology [27]. An OR significantly < 1 favored RA, whereas an OR significantly > 1 favored LA. All *P* values < 0.05 was considered statistically significant. Pooled estimates were calculated with the fixed-effect model (Mantel-Haenszel method) [28] if no significant heterogeneity was detected; otherwise, the random-effect model (DerSimonian-Laird method) was used [29]. The Cochrane chi-square test (Q) and inconsistency (I2) were used to evaluate the heterogeneity among studies.

## RESULTS

From our systematic literature review we identified 13 studies that compared RA and LA including 798 patients. 379 underwent to RA (cases group) and 419 to LA (controls group). 8 studies were prospective, but only one was a randomized controlled trial. 5 studies instead were retrospective. In Table 1 we showed studies characteristics, year and country of publication, period of study interval and level of evidence. We divided the variables analyzing in three groups: preoperative; operative and surgical outcomes. We considered also follow up and costs of RA but there was a lack of data regarding these aspects.

**Table 1:** Characteristics of eligible studies

Study	Country	Study interval	Study design	Level of Evidence
Morelli et al. (2016)	Italy	1994–2014	Retrospective	2b
Pahwa et al. (2015)	India	2010–2013	Retrospective	3b
Brandao et al. (2014)	USA	2004–2013	Retrospective	2b
Aliyev et al. (2013)	USA	2000–2012	Prospective	2b
You et al. (2013)	Korea	2009–2012	Retrospective	3b
Aksoy et al. (2013)	USA	2003–2012	Prospective	2b
Pineda-Solis et al. (2013)	USA	NA	Retrospective	3b
Agcaoglu et al. (2012)	USA	2000–2011	Prospective	2b
Agcaoglu et al. (2012)	2012	2009–2011	Prospective	2b
Karabulut et al. (2012)	USA	2008–2010	Prospective	2b
Brunaud et al. (2008)	France	1996–2005	Prospective	2b
Wu et al. (2008)	Taiwan	2003–2005	Prospective	2b
Morino et al. (2004)	Italy	2002	RCT	2a

### Demographics and preoperative characteristics

**Figure 2:**
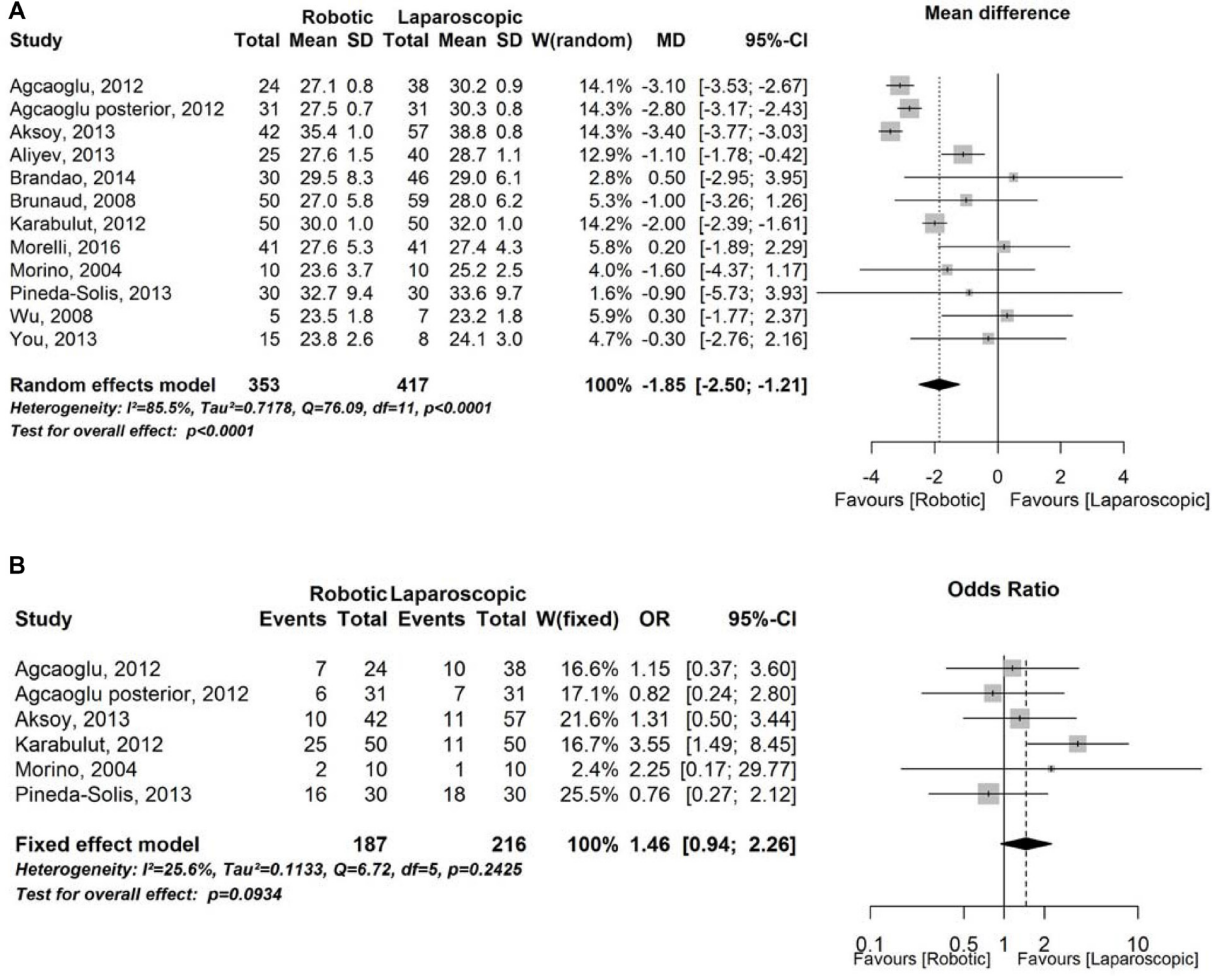
Demographics and preoperative characteristics (**A**) Forest plot representing analysis of Body Mass Index. CI = confidence interval; MD = mean difference; SD = standard deviation; W = Weight. (**B**) Forest plot representing analysis of previous surgery rate. CI = confidence interval; OR = Odds Ratio; W = Weight.

There were no significant differences between the two groups of patients respect to age, gender, laterality and tumor size. BMI instead was significant lower in the robotic group (Figure 2A). In robotic group we found patients with higher incidence of previous abdominal surgery (Figure 2B) but there was no significant difference.

### Operative variable

**Figure 3:**
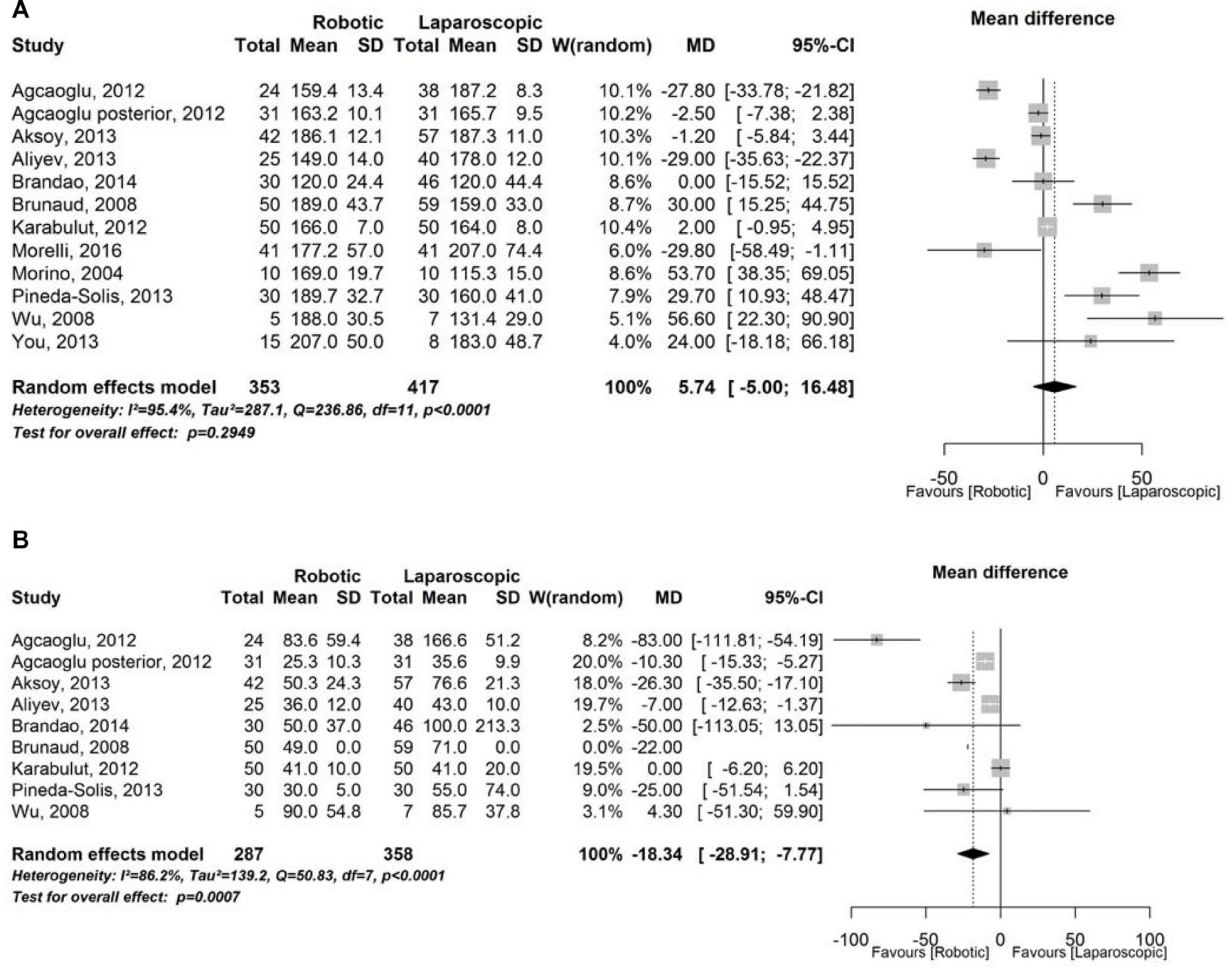
Operative variable (**A**) Forest plot representing analysis of operative time. CI = confidence interval; MD = mean difference; SD = standard deviation; W = Weight; (**B**) Forest plot representing analysis of estimated blood loss. CI = confidence interval; MD = mean difference; SD = standard deviation; W = Weight.

In this category we considered the surgical technique in the preferred approach among the two groups with no significant differences. The results from operative time demonstrated lower operative time for LA but there were no significant differences with robotic group (Figure 3A). RA instead showed a significant lower blood loss (Figure 3B).

### Surgical outcomes

**Figure 4:**
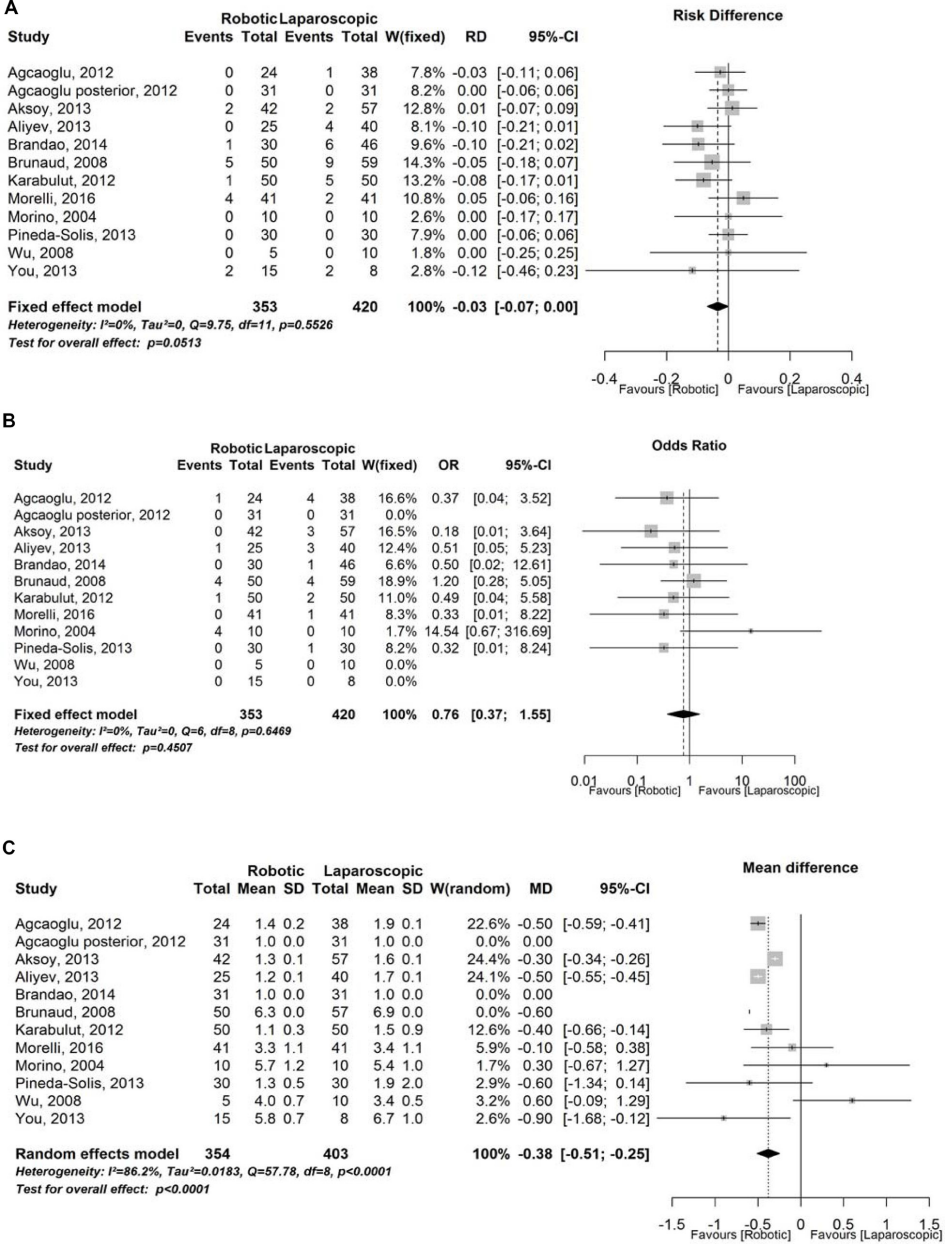
Surgical outcomes (**A**) Forest plot representing analysis of complication rate. CI = confidence interval; RD = Risk Difference; SD = standard deviation; W = Weight; (**B**) Forest plot representing analysis of conversion rate. CI = confidence interval; OR = Odds Ratio;W = Weight; (**C**) Forest plot representing analysis of length of hospital stay. CI = confidence interval; MD = mean difference; SD = standard deviation; W = Weight.

There were no differences in term of pathological results. Overall complications and conversion rate were expression of surgical safety and seemed to be favoring of RA but with no significant differences (Figure 4A–4B). Length of hospital stay was significant lower in robotic arm (Figure 4C). In this study we tried to analyze also follow-up and costs of different procedures, robotic and laparoscopic, but there was no systematic data compilation in eligible articles so we were not able to perform a rigorous meta-analysis.

## DISCUSSION

Since first LA in 1992 [1] endocrine surgeons developed interest for mini-invasive surgery thanks to better clinical outcomes, lower perioperative morbidity and mortality, shorter hospitalization and better cosmetic results [30]. In 1999 Piazza et al. [11] demonstrated the feasibility of robot-assisted adrenalectomy using ZEUS AESOP (Computer Motion, Inc., Santa Barbara, CA). For many years technology made numerous improvements in robotic platform with the advent of da Vinci system (Intuitive Surgical, Sunnyvale, CA, USA) that allowed worldwide diffusion of robotic surgery, initially for radical prostatectomy and later for several surgical procedures especially for those requiring advanced surgical skills like sutures and intracorporeal knotting [31]. Robotic platforms present numerous advantages like endo-wrist movements with 7 degrees of freedom, absence of surgeon related tremors and stereoscopic vision. Although LA is a safe and diffuse procedure among endocrine surgeons, we performed this systematic literature review with meta-analysis comparing laparoscopic and robotic approach. We considered particular deep location of adrenal loggia with theoretical maximum advantage of 3D vision system and endo-wrist movements. These characteristics could be reasonably used in complex patients (previous surgery, BMI > 30 kg/m2). The large diffusion of RA was clear from our literature research with several cases series in the last years. We found other two meta-analysis that compared RA and LA but these studies took in consideration initial limited experience in RA [32, 33] and obtained some different results. Furthermore, we had to consider the evolution of robotic platform and consequently the differences in surgical results. We did not found significant differences in demographics and preoperative characteristics except for BMI and history of previous surgery. We observed lower BMI and higher incidence of previous abdominal surgery in robotic group. From literature we knew that obesity represents an independent risk factor in adrenal surgery [34]. The most articles analyzed in this study were not randomized and the difference in BMI seemed to be a bias in patient selection because surgeons choosed patients who are generally fitter to facilitate robotic procedures. The only prospective randomized controlled trial by Morino et al [26] did not show significant difference regarding BMI in the two groups with a limited number of patients. Aksoy et al. [19] for the first time compared RA versus LA in obese patients (BMI 35.4 ± 1.0 in robotic group vs 38.8 ± 0.8 Kg/m^2^ in laparoscopic group, *p* = 0.01) with no difference in perioperative outcomes. Authors believed that these results underlined the difficulties with robotic approach of obtaining and manteining appropriate exposure due to patient habitus and malposition of robotic trocars. Authors overcame this difficulty by either using more additional trocars or moving the position of the first assistant port. On the contrary, Brunaud et al. [24] reported different results because the robotic approach offered several advantages in obese patients with no higher operative time when compared with LA. Surgical technique was comparable to laparoscopic surgery except for an additional trocar in RA [35, 36]. Agcaoglu et al. [23] performed a specific prospective study about posterior retroperitoneal adrenalectomy. Their hypothesis was that the two approaches, laparoscopic and robotic, would have had a similar operative outcome but that operative time would have been shorter with the robotic technique thanks to its more dexterous instrumentations. Statistical analysis showed similar operative time, with smaller (diameter < 6 cm) selected adrenal lesion in robotic group, and RA was shorter only after the 10th procedure such as an effect of surgeon's learning curve. Operative time, intraoperative blood loss and other complications and conversion rate were direct objective variables to evaluate the efficacy and safety of a new surgical technique. In a previous meta-analysis by Tang et al. [32] there was a significant difference in the operative time in favor of LA. Our results instead are agree with another study by Brandao et al. [33] that reported no statistically significant difference in this parameter. This evidence in our opinion was due to several different factors related to surgical team (robot setup and docking, resident surgeon or fellow). Agcaoglu et al. [21] reported a shorter operative time in RA in selected patients with large adrenal tumors. We described the controversies regarding the use of minimal invasive surgery in case of large adrenal masses because of technical limitations, longer operative time and increased blood loss [37, 38]. The critical difference between robotic and laparoscopic surgery was related to three-dimensional vision and wristed robotic instruments versus rigid laparoscopic devices. Karabulut et al. [22] focused their attention to time data on individual steps and skin-to-skin duration of procedures: on multivariate analysis they showed that RA performed by two staff surgeons was shorter than procedures with one staff surgeon and a fellow as first assistant with also fewer instruments changes and less need to clean the camera. Several studies reported that to keep the robot in a dedicated operating room and to do the other preparations of robotic platform during induction of anesthesia as well as initial laparoscopic phase of the procedure and the same learning curve of all members of surgical team reduced operative time. Brunaud et al. [24] observed no significant differences in operative time after the learning curve of 20 cases. On the other hand, Agcouglu et al. [21] reported a significant improvement in operative time after only the tenth procedure in the robot-assisted group. All eligible articles showed a significant reduction of estimated blood loss in RA due to stereoscopic vision and to more precise dissection plane when using robotic arms. Although this difference was statistically significant, it was probably not clinically relevant. There were no significant differences in terms of conversion and overall complications rate, related to specific pathological results (e.g. pheocromocytoma, adrenocortical carcinoma, ACC) and to general clinical conditions rather than to the surgical procedure itself. From analysis of eligible studies conversion rate was similar in the two group. In the literature conversion rate for robotic approach was between 0–40%, instead was 0–10.5% for laparoscopic group. The highest conversion rate was of 40% derived from first randomized study by Morino [26]. The reasons of conversion were malposition of robotic trocars, prolonged operative time and difficulties to obtain accurate hemostasis. This last observation realistically was related to the initial lack of advanced energy source with typical use of monopolar scissor or bipolar forceps. We positively believe that technological advances and new multi-use instruments development will improve dissection reducing operative time, bleeding and complications. Morelli et al. [14] reported two vascular lesions (one vena cava and one left renal vein damage) among intraoperative complications in RA for large adrenal tumors managed by using sutures without the necessity to convert to laparoscopic or open surgery. Length of hospital stay was significant shorter in RA when compared to LA. In accord with different authors we thought that an accurate robotic surgical dissection and reduction in blood loss improved the postoperative recovery of these patients. On the other side robotic approach registered a longer operative timeand additional trocars that were in contrast with a reduced hospital stay. The difference could hide an operator relating bias explained with the positive expectations from a new procedure and the medical staff focused on the early hospital discharge [38, 39]. Only few studies of this research reported a long-term follow up for their patients. This was due to treatment, in large part, of benign adrenal diseases [14, 16, 17, 21, 24]. Still today increased costs represent the real drawbacks of the robotic procedures. In their prospective randomized controlled trial Morino et al. [26] reported higher cost of the robotic procedure without including the initial cost to buy the da Vinci system. The increased costs were mainly due to the use of semi-disposable robotic instruments and longer operative time. Different studies [24, 19] calculated that the robotic procedures were 1.2–2.3 times more expensive than LA, but might take on a greater value in terms of marketing for the hospital. These authors concluded that capital and maintenance costs could be affordable at high-volume robotic surgery center reducing mean hospital stay and increasing the use of the robot by other surgical services.

## CONCLUSIONS

This study is a systematic literature review with meta-analysis, but we must consider some limitations. The studies reviewed are retrospective and prospective, but only one is a randomized controlled trial that shows initial data of limited number of patients. The use of retrospective studies increases the possibility of selection bias with doubts in interpreting results. On the other hand the authors have different surgical experience and this can reflect different outcomes. Most of the studies take into consideration only benign adrenal pathology with no long-term follow-up. In literature we find other reviews and meta-analysis regarding initial, limited experience with robotic adrenalectomy. The reason for a new meta-analysis is due to rapid evolving of robotic technology and increased experience of dedicated surgeon with improved clinical outcomes. This present meta-analysis includes 798 patients: 379 underwent to RA (cases group) and 419 treated with LA (controls group). Our aim is that to evaluate demographic characteristics, operative parameters and clinical outcomes between RA and LA. RA is a safe and feasible technique with reduced blood loss and shorter hospital stay than LA. Laparoscopic approach seems to be a more rapid technique when comparing to RA, although recent studies demonstrate a significant operative time reduction in RA with the learning curve improvement and the development of new surgical technology and advanced energy source.
